# Quantitative proteomics on the cerebrospinal fluid of hydrocephalus in neonatal bacterial meningitis

**DOI:** 10.3389/fped.2022.972032

**Published:** 2022-08-16

**Authors:** Juncao Chen, Weiben Huang, Hong Zhang, Xiangwen Peng, Jun Yang, Yong Yang, Jinzhen Su, Siyao Wang, Wei Zhou

**Affiliations:** ^1^Department of Neonatology, Guangzhou Women and Children’s Medical Centre, Guangzhou Medical University, Guangzhou, China; ^2^Department of Neonatology, The Fifth Affiliated Hospital of Southern Medical University, Guangzhou, China; ^3^Department of Neonatology, Dali Autonomous Prefecture Children’s Hospital, Dali, China; ^4^Department of Key Laboratory, Changsha Hospital for Maternal and Child Health Care, Changsha, China; ^5^Advanced Institute of Natural Sciences, Beijing Normal University at Zhuhai, Zhuhai, China; ^6^Department of Neonatology, Dongguan Maternal and Child Health Hospital, Dongguan, China

**Keywords:** quantitative proteomics, biomarkers, hydrocephalus, bacterial meningitis, differentially expressed proteins

## Abstract

**Objective:**

Hydrocephalus in bacterial meningitis (BM) is a devastating infectious neurological disease and the proteins and pathways involved in its pathophysiology are not fully understood.

**Materials and methods:**

Label-free quantitative (LFQ) proteomics analyses was used to identify differentially expressed proteins (DEPs) in cerebrospinal fluid (CSF) samples from infants with hydrocephalus and bacterial meningitis (HBM group, *N* = 8), infants with bacterial meningitis (BM group, *N* = 9); and healthy infants (N group, *N* = 11). Bioinformatics analysis was subsequently performed to investigate Gene Ontology (GO) functional annotation and Kyoto Encyclopedia of Genes and Genomes (KEGG) enriched signaling pathways of these DEPs. Six proteins (AZU1, COX4I1, EDF1, KRT31, MMP12, and PRG2) were selected for further validation *via* enzyme-linked immunosorbent assay (ELISA).

**Results:**

Compared with BM group and N group, HBM group had a higher whole CSF protein level (5.6 ± 2.7 vs. 1.7 ± 1.0 vs. 1.2 ± 0.5 g/l) and lower whole CSF glucose level (0.8 ± 0.6 vs. 1.8 ± 0.7 vs. 3.3 ± 0.8 mmol/l) (both *P* < 0.05). Over 300 DEPs were differentially expressed in HBM group compared with BM group and BM compared with N group, of which 78% were common to both. Cluster analysis indicated that the levels of 226 proteins were increased in BM group compared with N group and were decreased in HBM group compared with BM group. Bioinformatics analysis indicated the involvement of the cell adhesion, immune response and extracellular exosome signaling were significantly enriched in HBM compared with BM group and BM compared with N group. 267 DEPs were identified between HBM group with N group, KEGG analysis indicated that DEPs mainly involved in filament cytoskeleton and immune response. The ELISA results further verified that the expression levels of AZU1 were significantly different from among three groups (both *P* < 0.05).

**Conclusion:**

This is the first reported characterization of quantitative proteomics from the CSF of infants with HBM. Our study also demonstrated that AZU1 could be a potential biomarker for the diagnosis of hydrocephalus in bacterial meningitis.

## Introduction

Bacterial meningitis (BM) which is a serious infectious neurological disease frequently occurs in neonates and children. Infants have the highest incidence of BM in all age groups; according to population-based studies, the incidence of neonatal BM is estimated at 0.3 cases per 1000 in developed countries (1, 2). Despite advances in infant intensive care in recent decades, 20–50% of infants who survive BM in high-income countries still developed neurologic sequelae (3–5). These neurologic sequelae include hydrocephalus, subdural effusions, focal neurologic deficits, cerebrovascular abnormalities, hearing loss, cognitive impairment, and epilepsy. Hydrocephalus which is one of the most common complications of BM is the main factor of mortality (6, 7). Based on previous studies, most hydrocephalus in the neonatal period is related to restriction of the flow of cerebrospinal fluid (CSF) (8–10). Despite its high prevalence and mortality in the population, very little is known about the molecular mechanism of hydrocephalus in BM.

Cerebrospinal fluid that bathes the brain and spinal cord is the main component of the brain’s extracellular space. In addition, CSF contains proteins, enzymes, and a number of other physiologically important substances that reflect the composition of the brain and the physiological processes occurring in the central nervous system (11–13). Moreover, CSF proteins can be used to predict the outcome of BM and serve as a tool for the diagnosis of BM. Unfortunately, there were no previous reports about the protein profiling of CSF in infants with hydrocephalus and bacterial meningitis (HBM). Consequently, in this study, our team applied label-free quantitative mass spectrometry (LFQ-MS) based proteomics to the analysis of CSF samples from infants with (HBM infants), infants with (BM infants), and healthy infants (N infants). The aim of the present work was to compare the differentially expressed proteins (DEPs) of CSF among the three groups mentioned above and to provide new insights into the pathogenesis involved in the hydrocephalus in BM.

## Materials and methods

### Patient samples

The study protocols were approved by the research ethical committee of the Guangzhou Women and Children’s Medical Centre of Guangzhou Medical University. All parents were fully informed and signed written informed consent in this study. The infants were categorized into the following groups: N group, BM group, and HBM group.

All infants underwent lumbar puncture (LP) at Guangzhou Women and Children’s Medical Centre of Guangzhou Medical University (2019–2021). Infants with chromosomal aberrations, genetic syndromes, and intracranial hemorrhage were excluded. In this study, the inclusion criteria for neonatal BM included one or more of the following: (a) isolation of a bacterial pathogen from CSF culture; (b) isolation of the same bacterial pathogen from blood drawn simultaneously at two different sites, with classic characteristics of BM (CSF pleocytosis defined as absolute leukocyte count ≥20 cells/mm^3^, with a decreased glucose level ≤2.2 mmol/l and an elevated protein concentration ≥1 g/l); and (c) no pathogen isolated from either CSF or blood, with clinical symptoms and classic characteristics of BM (CSF pleocytosis defined as absolute leukocyte count ≥20 cells/mm^3^, with a decreased glucose level ≤2.2 mmol/l and an elevated protein concentration ≥1 g/l) (14). Cranial ultrasound and magnetic resonance imaging (MRI) were performed on all infants with BM to diagnose hydrocephalus. Hydrocephalus was defined as ventriculomegaly with Evan’s ratio (maximal width of frontal horns/maximal width of inner skull) >0.30 and/or lateral ventricles with ventricular width >97 th centile or anterior horn width >6 mm (15).

Lumbar puncture CSF was collected between October 2019 and May 2021. Each CSF sample was collected *via* a syringe into a polypropylene sample collection tube. The total CSF (approximately 1–2 ml) was retained and centrifuged at 4°C for 10 min at 2,000 × *g*, frozen and then stored at −80°C until analysis.

### Cerebrospinal fluid protein extraction and quantification

Cerebrospinal fluid samples were reduced with 6 M guanidine hydrochloride solution containing 1% protease inhibitor, placed in an ice bath and ultrasonicated for 15 min. Samples were then reduced with 20 mmol/l dithiothreitol (DTT) for 1.5 h at 56°C, subsequently alkylated with 50 mmol/l iodoacetamide (IAA, Sigma–Aldrich) for 30 min at room temperature protected from light. Samples were further digested overnight at 37°C in ammonium bicarbonate (ABC) with trypsin. After the first digestion, additional trypsin was added at 37°C for 12 h. To stop the proteolytic reaction, trifluoroacetic acid (TFA) was added, followed by incubation for 45 min at 37°C. Peptides were dissolved in 80% acetonitrile (ACN), and 0.1% formic acid for 120 min and then injected into an Eksigent-nano-HPLC system (Sciex, Framingham, MA, United States) by an autosampler and separated on a C18 desalting column (75 μm × 200 mm, AQ, 1.9 μm).

### Liquid chromatography–tandem mass spectrometry analysis

Liquid chromatography–tandem mass spectrometry (LC–MS/MS) analysis was performed on an EASY-nLC 1200-nanometer UHPLC system coupled with a Q Exactive TM Plus hybrid quadrupole-orbitrap mass spectrometer (Thermo Fisher Scientific). The parameters of the mass spectrometer were set as follows: the full scan range of the MS was m/z 350-1700, the resolution of detection of intact peptides was set at 70,000 with a dynamic first mass, the max injection time was 100 ms, the fragmentation energy mode was dynamic fragmentation mode with higher energy collisional dissociation (HCD), and a dynamic exclusion of 60 s was used.

### Protein identification, quantification, and data analysis

Raw data generated by MS were quantitatively analyzed by Proteome Discoverer 2.4 software (PD2.4, Thermo Fisher Scientific, Waltham, MA, United States) along with common contaminants using the MaxQuant platform. The database is UniProtKB (2015_04, 42 121). The search parameters of PD2.4 software were set as follows: (a) trypsin was set as a specific enzyme allowing up to 2 missing cleavages; (b) carbamidomethylation of cysteine was a fixed modification, and the variable modification was oxidation modification of methionine and acetylation modification of the N-terminal; and (c) the mass precursor tolerance was set to 10 ppm, and 0.5 Da was set for fragment ions.

### Bioinformatics analysis

Statistical analysis of data was performed in three steps: (1) N group vs. BM group; (2) BM group vs. HBM group; and (3) HBM group vs. N group. One-way ANOVA was applied to detect the significant DEPs, and the filter criterion for proteins with significant differences in quantification between the groups was | Log_2_ fold change| > 1 and *P* < 0.05. Gene Ontology (GO) functional analysis was performed for functional analysis of the DEPs, and proteins were classified into three major GO categories: biological process (BP), molecular function (MF), and cellular component (CC). Kyoto Encyclopedia of Genes and Genomes (KEGG) analysis was performed to further explore the pathway analysis of the identified proteins. Potential protein-protein interaction network analysis was performed using STRING DB software. The volcano plot analysis was performed for the identified proteins. Cluster analysis was performed to further explore the change rule of DEPs among the three groups. Clustering analysis was performed using Graphical Proteomics Data Explorer (GProX).

### Enzyme linked immunosorbent assays

The expression levels of DEPs (AZU1, COX4I1, EDF1, KRT31, MMP12, and PRG2) in CSF samples among the three groups were measured using double-antibody sandwich ELISA kits (purchased from Jiangsu Jing Mei Biological Technology Co., Ltd., China). The operation process of ELISA assay followed the supplier’s protocol. The human matrix proteoglycan 2 (PRG2) ELISA kit (JM-04983H1, Jingmei, China), human matrix keratin 3 (KRT3) ELISA kit (JM-5519H1, Jingmei, China), human matrix azurocidin 1 (AZU1) ELISA kit (JM-5490H1, Jingmei, China), human matrix cytochrome C oxidase subunit 4I1 (COX4I1) ELISA kit (JM-1164H1, Jingmei, China), human matrix matrix metallopeptidase 12 (MMP12) ELISA kit (JM-04983H1, Jingmei, China), and human matrix endothelial differentiation related factor 1 (EDF1) ELISA kit (JM-5462H1, Jingmei, China) were used. The absorption value (optical density value) was measured at A450 using a microplate spectrophotometer (Bio-Rad), and the expression level was calculated using standards.

### Statistical analysis

Data were analyzed by using SPSS version 22. Continuous variables were statistically analyzed by one-way ANOVA or the Mann–Whitney *U*-test, and differences were expressed as the means ± SD. χ^2^ tests were used for categorical variables that were presented as numbers and percentages. *P* < 0.05 was regarded as indicating a significant difference.

## Results

### Characterization of the cerebrospinal fluid content among the three groups

A total of 28 infants were included in our study: 11 N infants, 9 BM infants, and 8 HBM infants. Table 1 showed the basic characteristics of the infants, in which 10 infants (58.8%) had positive pathogen cultures and 7 infants (41.2%) had a clinical diagnosis of BM with negative pathogen cultures. No significant difference was observed in gestational age or sex among the three groups. Compared with BM group and N group, HBM group had a higher whole CSF protein level (5.6 ± 2.7 vs. 1.7 ± 1.0 vs. 1.2 ± 0.5 g/l), higher peripheral blood C-reactive protein (CRP) level (93.2 ± 56.2 vs. 47.2 ± 36.0 vs. 4.1 ± 4.9 mg/l), higher CSF white blood cell count (467.2 ± 544.3 vs. 320.0 ± 411.5 vs. 4.1 ± 6.4 10^∧^6/L) and lower whole CSF glucose level (0.8 ± 0.6 vs. 1.8 ± 0.7 vs. 3.3 ± 0.8 mmol/l) (all *P* < 0.05) (Table 1).

**TABLE 1:** Baseline demographical and routine laboratory characteristics of CSF content among three groups.

Patient’s parameters	N group	BM group	HBM group	*P*-value
Gestational age (weeks)	35.0 (5.3)	37.3 (3.6)	34.7 (5.2)	0.459
Gender (female/male)	4:7	3:6	2:6	0.868
Serum CRP (mg/L)	4.1 (4.9)	47.2 (36.0)	93.0 (56.2)	<0.01
Positive CSF bacterial culture	0 (0)	4 (44.4)	6 (75.0)	0.201
Whole CSF WBC (10^∧^6/L)	4.1 (6.4)	320.0 (411.5)	467.2 (544.3)	0.032
Whole CSF protein (g/L)	1.2 (0.5)	1.7 (1.0)	5.6 (2.7)	<0.01
Whole CSF chlorine (g/L)	124.4 (6.3)	122.8 (3.5)	120.6 (10.0)	0.507
Whole CSF glucose (mmol/l)	3.3 (0.8)	1.8 (0.7)	0.8 (0.6)	<0.01
CSF neutrophil, (%)	1.6 (4.5)	52.8 (24.4)	60.6 (14.8)	<0.01

CRP, C-reactive protein; CSF, cerebrospinal fluid; WBC, white blood cell.

### Identification of significant proteins by label-free quantitative analysis

**FIGURE 1:**
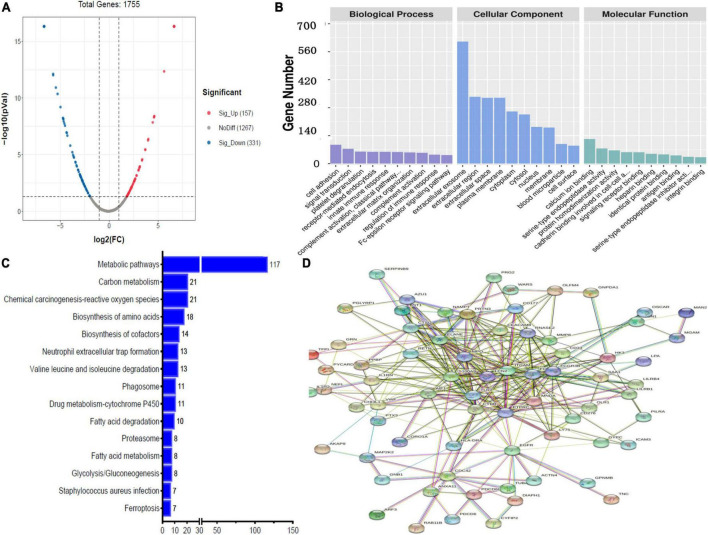
Bioinformatics analysis of the differentially expressed proteins (DEPs) between hydrocephalus and bacterial meningitis (HBM) group and bacterial meningitis (BM) group. **(A)** Volcano plot showing DEPs. **(B)** Gene Ontology (GO) analysis. **(C)** Kyoto Encyclopedia of Genes and Genomes (KEGG) analysis. **(D)** STRING protein-protein network enrichment analysis.

Label-free quantitative analysis was performed on the CSF samples between HBM group and BM group to identify DEPs. A total of 1755 proteins were identified. Compared to BM group, 157 proteins were upregulated and 331 proteins were downregulated in the HBM group; a volcano plot is shown in Figure 1A. The top 5 upregulated proteins were PRG2, IGLV3-21, KRT31, AZU1, and UGGT1, and the top 5 downregulated proteins were H1-0, FTL, COX4I1, GSTM1, and PRSS1 (Table 2). The STRINGdb database was used to search for the identified proteins above thus to observe the interactions between DEPs (Figure 1D).

**TABLE 2:** Ten significant proteins were identified between HBM group and BM group (A), BM group and N group (B), HBM group and N group (C) by label-free quantification.

	Majority protein IDs	Protein name	Gene name	Fold-change	Adj. *P*-value
A	P13727	Proteoglycan 2	*PRG2*	49.435	<0.0001
	P80748	Immunoglobulin Lambda variable 3–21	*IGLV3-21*	24.600	<0.0001
	Q15323	Keratin 31	*KRT31*	24.455	<0.0001
	P20160	Azurocidin 1	*AZU1*	24.093	<0.0001
	Q9NYU2	UDP-glucose glycoprotein glucosyltransferase 1	*UGGT1*	22.020	<0.0001
	P07305	H1.0 linker histone	*H1-0*	0.019	<0.0001
	P02792	Ferritin light chain	*FTL*	0.019	<0.0001
	P13073	Cytochrome C Oxidase subunit 4I1	*COX4I1*	0.023	<0.0001
	P09488	Glutathione S-Transferase Mu 1	*GSTM1*	0.026	<0.0001
	P07477	Serine protease 1	*PRSS1*	0.032	<0.0001
B	Q14749	Glycine N-Methyltransferase	*GNMT*	22.109	<0.0001
	P13073	Cytochrome C Oxidase Subunit 4I1	*COX4I1*	21.811	<0.0001
	P08397	Hydroxymethylbilane Synthase	*HMBS*	21.411	<0.0001
	P32754	4-Hydroxyphenylpyruvate Dioxygenase	*HPD*	20.486	<0.0001
	O75891	Aldehyde Dehydrogenase 1 family member L1	*ALDH1L1*	17.681	<0.0001
	P01266	Thyroglobulin	*TG*	0.025	<0.0001
	P60900	Proteasome 20S subunit alpha 6	*PSMA6*	0.061	<0.0001
	P80748	Immunoglobulin Lambda variable 3–21	*IGLV3-21*	0.073	<0.0001
	P0DJI8	Serum amyloid A1	*SAA1*	0.115	<0.0001
	P11137	Microtubule associated protein 2	*MAP2*	0.127	<0.0001
C	O75888-1	Tumor necrosis factor ligand superfamily member 13	TNFRSF13	14.433	<0.0001
	P05109	S100 calcium binding protein A8	*S100A8*	11.156	<0.0001
	P51668	Ubiquitin conjugating enzyme E2 D1	*UBE2D1*	10.996	<0.0001
	P20160	Azurocidin 1	*AZU1*	10.833	<0.0001
	P20851	Complement component 4 binding protein beta	C4BPB	10.129	<0.0001
	P10909-3	Isoform 3 of clusterin	*CLU*	0.027	<0.0001
	P05787	Keratin 8	*KRT-8*	0.05	<0.0001
	Q9NZT1	Calmodulin-like protein 5	*CALML5*	0.062	<0.0001
	P02545	Lamin A/C	*LMNA*	0.067	<0.0001
	Q5T749	Keratinocyte pro-line rich protein	*KPRP*	0.069	<0.0001

**FIGURE 2:**
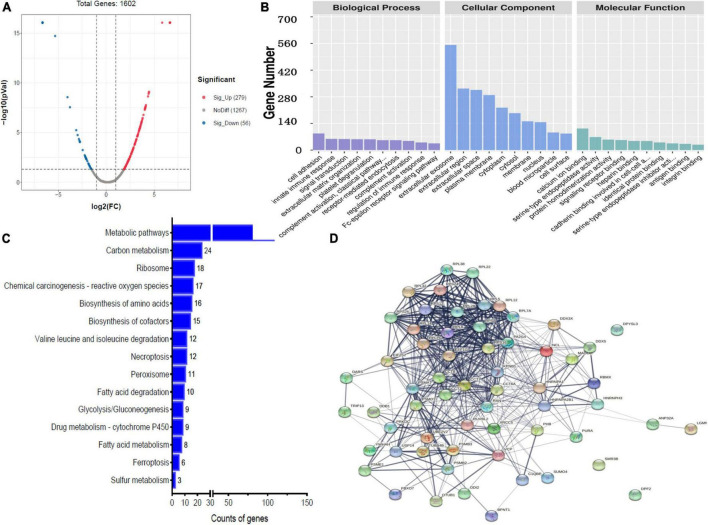
Bioinformatics analysis of the differentially expressed proteins (DEPs) between bacterial meningitis (BM) group and N group. **(A)** Volcano plot showing DEPs. **(B)** Gene Ontology (GO) analysis. **(C)** Kyoto Encyclopedia of Genes and Genomes (KEGG) analysis. **(D)** STRING protein-protein network enrichment analysis.

A total of 335 proteins were found to be significantly differentially expressed between BM group and N group, including 56 upregulated and 279 downregulated proteins. A volcano plot showing statistically significant DEPs was constructed (Figure 2A). The top 5 upregulated proteins were GNMT, COX4I1, HMBS, HPD, and ALDH1L1, and the top 5 downregulated proteins were TG, PSMA6, IGLV3-21, SAA1, and MAP2 (Table 2). The STRINGdb database was used to search for the identified proteins above thus to observe the interactions between DEPs (Figure 2D).

**FIGURE 3:**
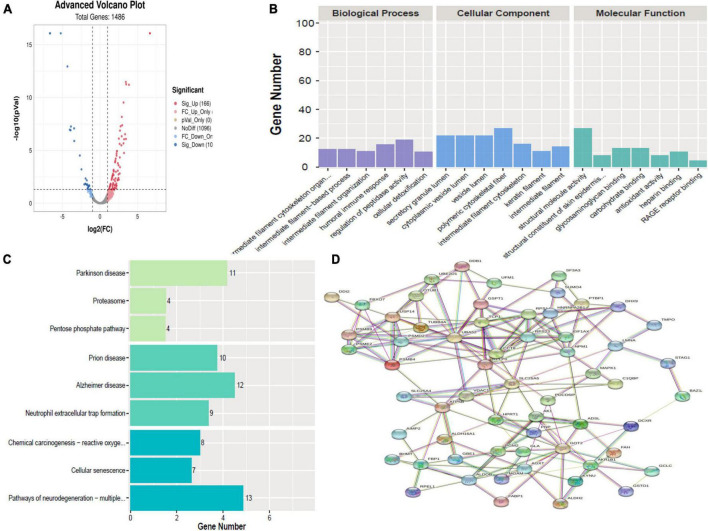
Bioinformatics analysis of the differentially expressed proteins (DEPs) between hydrocephalus and bacterial meningitis (HBM) group and N group. **(A)** Volcano plot showing DEPs. **(B)** Gene Ontology (GO) analysis. **(C)** Kyoto Encyclopedia of Genes and Genomes (KEGG) analysis. **(D)** STRING protein-protein network enrichment analysis.

A total of 267 proteins were found to be significantly differentially expressed between HBM group and N group, including 166 upregulated and 101 downregulated proteins. A volcano plot showing statistically significant DEPs was constructed (Figure 3A). The top 5 up-regulated proteins TNFRSF13, S100A8, UBE2D1, AZU1, and C4BPB, and the top 5 downregulated proteins were CLU, KRT8, CALML5, LMNA, and KPRP (Table 2). The STRINGdb database was used to search for the identified proteins above thus to observe the interactions between DEPs (Figure 3D).

### Gene ontology analysis of significant proteins

Gene ontology analysis was performed to gain more insight into the biological significance of the DEPs. A comparison of HBM group with BM group revealed that the top 5 BPs were cell adhesion, signal transduction, platelet degranulation, receptor-mediated endocytosis and innate immune response; the top 5 CCs were extracellular exosome, extracellular region, extracellular space, plasma membrane, and cytoplasm; and the top 5 MFs were calcium ion binding, serine-type endopeptidase activity, protein homodimerization activity, cadherin binding involved in cell–cell adhesion, and signaling receptor binding (Figure 1B).

Comparing BM group with N group revealed that the top 5 BPs were cell adhesion, innate immune response, signal transduction, extracellular matrix organization and platelet degranulation; the top 5 CCs were exosome, extracellular region, extracellular space, plasma membrane, and cytoplasm; and the top 5 MFs were calcium ion binding, serine-type endopeptidase activity, protein homodimerization activity, signaling receptor binding, and heparin binding (Figure 2B).

Comparing HBM group with N group revealed that the top 5 BPs were intermediate filament cytoskeleton organization, intermediate filament-based process, intermediate filament organization, humoral immune response, regulation of peptidase activity, and cellular detoxification; the top 5 CCs were polymeric cytoskeletal fiber, secretory granule lumen, cytoplasmic vesicle lumen, vesicle lumen, and intermediate filament cytoskeleton; and the top 5 MFs were structural molecule activity, structural constituent of skin epidermis, glycosaminoglycan binding, carbohydrate binding, and antioxidant activity (Figure 3B).

As shown in Figures 1B, 2B, most enriched Go terms in HBM group compared with BM group and BM group compared with N group were same.

### Kyoto encyclopedia of genes and genomes analysis of significant proteins

In a comparison of HBM group with BM group, KEGG pathway analysis implied that DEPs were primarily involved in five statistically significant pathways (*P* < 0.05), including metabolic pathways (*P* = 2.59E-22), carbon metabolism (*P* = 1.03E-09), chemical carcinogenesis–reactive oxygen species (*P* = 5.10E-05), biosynthesis of amino acids (*P* = 1.08E-10), and biosynthesis of cofactors (*P* = 4.96 E-04) (Figure 1C).

A comparison of BM group with N group, KEGG pathway analysis showed that DEPs were primarily involved in five statistically significant pathways (*P* < 0.05), including: metabolic pathways (*P* = 5.27E-22), carbon metabolism (*P* = 4.17E-13), ribosomes (*P* = 2.52E-08), chemical carcinogenesis–reactive oxygen species (*P* = 9.72E-04), and biosynthesis of amino acids (*P* = 1.74E-09) (Figure 2C).

A comparison of HBM group with N group, KEGG pathway analysis showed that DEPs were primarily involved in five statistically significant pathways (*P* < 0.05): Parkinson’s disease (*P* = 1.27E-06), neutrophil extracellular trap formation (*P* = 4.00E-06), Alzheimer’s disease (*P* = 7.53E-06), Prion disease (*P* = 1.13E-05), and pathways of neurodegeneration–multiple diseases (*P* = 1.32E-05) (Figure 3C).

As shown in Figures 1C, 2C, most enriched KEGG terms in HBM group compared with BM group and BM group compared with N group were same.

### Cluster analysis

**FIGURE 4:**
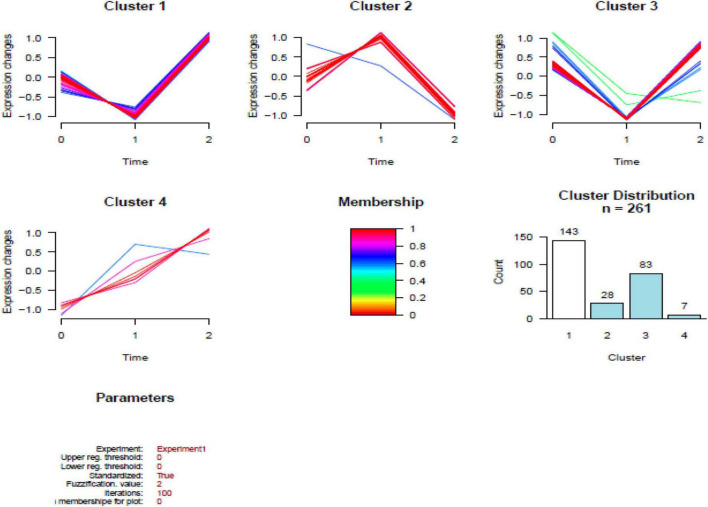
Results of the cluster analysis of the differentially expressed proteins (DEPs) among three groups. Cluster 1: Proteins down-regulated in infants with bacterial meningitis (BM) group, upregulated in infants with hydrocephalus and bacterial meningitis (HBM) group (higher than N group). Cluster 2: Proteins upregulated in infants with BM group, downregulated in HBM group. Cluster 3: Proteins downregulated in infants with BM group, upregulated in HBM group (not significantly different from N group). Cluster 4: Proteins up-regulated in BM group and HBM group. The scale is given by | Log_2_ fold change|. 0: N group; 1: BM group; 2: HBM group.

In total, 261 common DEPs were identified both in HBM group compared with BM group and BM group compared with N group; the levels of 7 proteins (PSMA7, PREP, PSMB2, VAT1, RETN, FBP1, and WARS1) were increased both in ABM group compared with BM group and in BM group compared with N group; the levels of 8 proteins (IGKV2-40, PPM1G, TG, IGLV2-14, MGP, UQCR1, TFG, and KRT74) were decreased both in HBM group compared with in BM group and BM group compared with N group; the levels of 246 proteins were increased in BM group compared with N group and were decreased in HBM group compared with BM group (Figure 4).

### Validation of identified proteins by enzyme-linked immunosorbent assay

**FIGURE 5:**
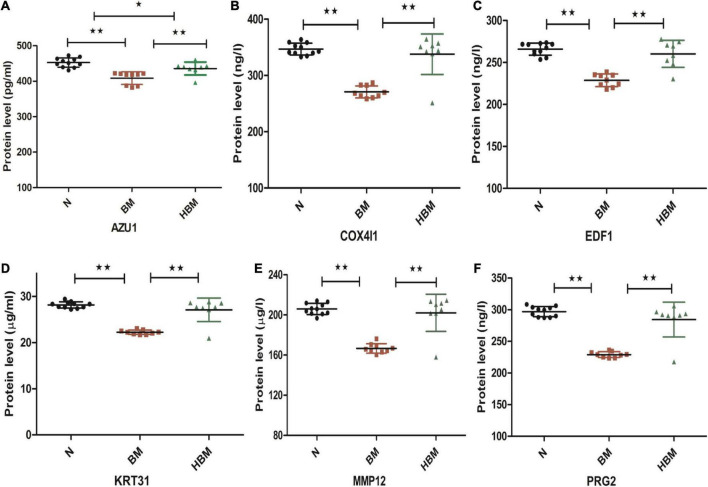
Enzyme-linked immunosorbent assay (ELISA) validation of AZU1, COX4I1, EDF1, KRT31, MMP12, and PRG2 proteins among N, bacterial meningitis (BM), and hydrocephalus and bacterial meningitis (HBM) groups. **(A-F)** Comparison of AZU1, COX4I1, ED1F, KRT31, MMP12, and PRG2 proteins in the three groups. *P < 0.05 and **P < 0.01.

Six differential proteins were selected to be further identified by ELISA. The expression levels of AZU1 in the hydrocephalus group were significantly different from these in the other groups. The expression levels of PRG2, MMP12, KRT31, COX4I1, and EDF1 were significantly upregulated in HBM group compared to BM group and were significantly downregulated in BM group compared to N group, but these five proteins were not significantly different between the HBM group and N group (Figure 5).

## Discussion

In this study we characterized the quantitative proteomics of CSF to investigate disease processes occurring due to hydrocephalus in neonatal BM. The main findings of our study provided a global view of the complex processes occurring in the CNS in infants with HBM. To our knowledge, this is the first comprehensive analysis to CSF proteomics in hydrocephalus in neonatal BM.

The pathogenesis of hydrocephalus in BM remains unclear. To better understand molecular mechanism of hydrocephalus, it is very important to clarify protein characteristics leading to disease emergence and progression. However, the pathogenesis of hydrocephalus in BM is poorly understood due to the lack of an appropriate experimental model and the challenge of sampling the site of disease. A significant shift in the CSF constituents and proteome can reflect cellular events in the brain, but there have been no reports about hydrocephalus in BM (16, 17). Previous studies have identified quantitative proteomics signatures in post-hemorrhagic hydrocephalus and idiopathic normal pressure hydrocephalus, these finding suggests that TLR4-NF-κB, mTOR, PDGFRα signaling and other pathways play an important role in hydrocephalus (18–21). A recent study demonstrated that STAT1 signaling plays an important role in hydrocephalus in John Cunningham polyomavirus (JCPyV) encephalopathy (22). However, these signatures were not applied to hydrocephalus in BM; the proteomics characteristics in our study were different from those in these studies, which indicated that hydrocephalus in BM has a unique pathophysiological mechanism.

Once the microbes enter the CSF, they proliferate and induce the release of proinflammatory proteins that cause pleocytosis. Previous studies demonstrated that increased protein levels (2.5 g/l) and CSF total cell counts in the CSF were risk factors for hydrocephalus in meningitis (23, 24). Our study also showed that HBM group had an upregulated CSF protein levels (5.6 ± 2.7 vs. 1.7 ± 1.0 g/l) and increased white blood cell count (467.2 ± 544.3 vs. 320.0 ± 411.5 10^∧^6/L) compared to those in the BM group.

Many brain cells can produce cytokine and pro-inflammatory proteins when bacterial enter the CSF, these pro-inflammatory proteins include reactive oxygen species (ROS), nitric oxide, peroxynitrite, matrix metalloproteinases (MMPs), platelet activating factor, calcium, etc (25, 26). A comparison of BM group with N group, GO analysis showed that the signal pathways that involve these pro-inflammatory proteins in active states in our data. HBM group had a higher white blood cell count in CSF than BM group in our study, and we speculated that a stronger inflammatory response may be found in HBM group. However, cluster analysis showed that most DEPs returned to normal levels in the HBM group. The interval from the first symptom of BM to the diagnosis of hydrocephalus ranged from 5 days to 4 weeks (27). So our data reveal the strong host response to infection in the neonatal brain and it can clear inflammatory proteins in a short time. Pleocytosis, pro-inflammatory and toxic compounds may block CSF circulation and increase blood-brain-barrier (BBB) permeability, and then lead to hydrocephalus (28–30). Previous studies demonstrated that the change of cell adhesion, cell tight junctions and cell cytoskeleton were related with BBB permeability (18, 21, 25); our study also observed the changes of these signaling pathways in GO analysis.

In the study, KEGG annotation suggested that Parkinson’s disease, Alzheimer’s disease, Prion disease, and pathways of neurodegeneration–multiple diseases were the enriched signaling pathways between HBM group and N group, which were in line with a previous proteomic study using a piglet model (31). 12 proteins (ATP5F1B, CALML5, PSMD2, PSMB4, PPP3CB, SLC39A10, SLC39A10, SLC25A4, SLC25A5, TUBB4A, TUBA1A, VDAC1) are co-exist in pathways of the above four neurological disease; this suggests that there may be a common mechanism between these neurological diseases and hydrocephalus in BM. But the role of these proteins in process of hydrocephalus in BM need to be further investigated.

AZU1 is an antimicrobial protein secreted by neutrophils that acts as a chemoattractant for monocytes and macrophages and a permeabilizer of vascular endothelial cells (32, 33). Previous findings showed that AZU1 plays an important role in encephalitis and Alzheimer’s disease, and can be used as a marker of immune response in these two diseases (34–36). In our study, AZU1 levels in CSF from the hydrocephalus group were significantly different from these in the other two groups, this indicating that AZU1 may be a potential protein biomarker for hydrocephalus in BM. PRG2, MMP12, and COX4I1 are related to neurological function, and previous studies proved that MMPs were biomakers of BM (37–40); however, these proteins were not significantly differed between the hydrocephalus group and healthy groups in our study. Previous study demonstrated that MMP12 was expressed early following inflammatory response with highest expression levels at 8 h and lower expression levels at 4 and 8 days (40, 41). EDF1 and COX4I1 are known to play an important role in regulating mitochondrial oxygen production, and ROS levels significantly spiked at 1.5 h after bacterial infection along with increased levels of EDF1 and COX4I1; but decreased levels of COX4I1 can induced host defense against *Listeria* by triggering mitochondrial ROS (42, 43). Therefore, changes in these four proteins in HBM group didn’t indicate the recovery of disease, it might be the manifestation of later host response (hydrocephalus may develop 1 or 2 weeks later after diagnosis with BM). But the role of these proteins in pathogenic process of hydrocephalus in BM need to be further investigated.

In summary, this study obtained a comprehensive overview of the proteins in CSF derived from infants with HBM by proteomic analysis, and this is the first shotgun proteomic survey reported in infants with HBM. However, there are still some limitations in our study. First, the sample size was relatively small. Second, the number of identified proteins using ELISA was not large, we finally identified only one candidate biomarker. Nevertheless, this untargeted proteomics study not only provided information on the pathogenic processes leading to hydrocephalus in BM but also identified a number of promising CSF proteins that warrant further validation in large prospective cohorts.

## Data availability statement

The raw data supporting the conclusions of this article will be made available by the authors, without undue reservation. The data presented in this study are deposited in the human_20210122 repository (Uniprot), accession number: 26517.

## Ethics statement

The studies involving human participants were reviewed and approved by the research Ethical Committee of the Guangzhou Women and Children’s Medical Centre of Guangzhou Medical University. Written informed consent to participate in this study was provided by the participants’ legal guardian/next of kin.

## Author contributions

JC, WH, HZ, and XP conceived and designed the experiments. JC, JY, YY, and JS performed the experiments. JC, WH, HZ, XP, and WZ analyzed the data. JC, JY, JS, and SW contributed to reagents the materials and analysis the tools. JC, WH, HZ, XP, YY, and WZ wrote the manuscript. All authors contributed to the manuscript’s critical revision and read and approved the submitted version.
