# Corrigendum: Quantitative proteomics on the cerebrospinal fluid of hydrocephalus in neonatal bacterial meningitis

**DOI:** 10.3389/fped.2023.1273209

**Published:** 2023-08-30

**Authors:** Juncao Chen, Weiben Huang, Hong Zhang, Xiangwen Peng, Jun Yang, Yong Yang, Jinzhen Su, Siyao Wang, Wei Zhou

**Affiliations:** ^1^ Department of Neonatology, Guangzhou Women and Children’s Medical Centre, Guangzhou Medical University, Guangzhou, China; ^2^ Department of Neonatology, The Fifth Affiliated Hospital of Southern Medical University, Guangzhou, China; ^3^ Department of Neonatology, Dali Autonomous Prefecture Children’s Hospital, Dali, China; ^4^ Department of Key Laboratory, Changsha Hospital for Maternal and Child Health Care, Changsha, China; ^5^ Advanced Institute of Natural Sciences, Beijing Normal University at Zhuhai, Zhuhai, China; ^6^ Department of Neonatology, Dongguan Maternal and Child Health Hospital, Dongguan, China

**Keywords:** quantitative proteomics, biomarkers, hydrocephalus, bacterial meningitis, differentially expressed proteins

A Corrigendum on Quantitative proteomics on the cerebrospinal fluid of hydrocephalus in neonatal bacterial meningitis By Chen J, Huang W, Zhang H, Peng X, Yang J, Yang Y, Su J, Wang S and Zhou W (2022). Front. Pediatr. 10:972032. doi: 10.3389/fped.2022.972032

**Incorrect Funding**

In the published article, there was an error in the Funding statement. The project number for the basic Research Project of Guangzhou Science and Technology Bureau was incorrectly displayed as “2210124-03”. The correct Funding statement appears below.

**FUNDING**

This article was supported by Research foundation of Guangzhou Women and Children's Medical Center for Clinical Doctor and Basic research program and applied basic Research Project of Guangzhou Science and Technology Bureau (202102010329).

The authors apologize for this error and state that this does not change the scientific conclusions of the article in any way. The original article has been updated.

